# Influence of Waste Tire Rubber Particle Size and Content on Mechanical Properties and Energy Dissipation of R-CTB

**DOI:** 10.3390/ma19091676

**Published:** 2026-04-22

**Authors:** Jie Wang, Yuanfan Liu, Kun Wang, Yan Li, Jianxin Fu

**Affiliations:** School of Resources and Safety Engineering, University of Science and Technology Beijing, Beijing 100083, China; wok-en-up@outlook.com (K.W.); d202510069@xs.ustb.edu.cn (Y.L.); fjx@ustb.edu.cn (J.F.)

**Keywords:** rubber particles, cemented tailings backfill, DIC, SEM, fractal dimension

## Abstract

To achieve the resource utilization of waste tires and improve the mechanical performance of cemented tailings backfill, rubber–cemented tailings backfill (R-CTB) specimens were prepared with four rubber particle sizes (20-, 40-, 60-, and 80-mesh) and four contents (2%, 4%, 6%, and 8%). A 0% rubber control group was introduced to address the lack of quantitative comparison. Uniaxial compression, digital image correlation (DIC), and scanning electron microscopy (SEM) were used to study mechanical behavior, energy evolution, and microstructural characteristics at 7 and 28 days. Results indicate that strength and elastic modulus first increase then decrease with particle size and decrease with content rise. Compared with the control group, R-CTB shows lower strength but significantly higher ductility and energy dissipation. Finer particles cause strain localization; higher content and finer size increase pores and weaken interfaces. Rubber incorporation transforms failure from brittle to ductile, providing a basis for engineering application.

## 1. Introduction

With the advancement of global industrialization and the growth in trade volume driven by population expansion, the generation of waste tires has increased dramatically, becoming an urgent environmental problem that needs to be addressed immediately [1,2,3]. According to relevant statistical data, the annual global production of waste tires reached 1.5 billion tons in 2020 and is projected to reach 1.2 billion tons by 2040 [4,5]. As the quantity of waste tires continues to rise, the traditional disposal methods mainly include stockpiling, landfilling, and incineration [6,7]. Stockpiling and landfilling not only occupy a large amount of land resources but also pose a serious threat to the ecological environment and human health due to the non-biodegradable nature, flammability, and tendency to release harmful substances. Incineration generates a large number of harmful gases, which poses a great threat to air quality [8,9]. Therefore, exploring effective recycling and resource utilization of waste tires has become a major issue in the field of environmental protection.

Waste tires are mainly composed of rubber, which can be crushed into smaller fragments during mechanical recycling to produce rubber particles [10,11]. Rubber, characterized by high ductility and low elastic modulus, can enhance the ductility, energy absorption capacity, and impact resistance of cement-based materials [6,12]. Against this background, cement-based concrete incorporating waste tire rubber particles, as an innovative approach for waste recycling and resource utilization, has gradually attracted the attention of relevant scholars.

Al-Subari et al. [13] incorporated waste tire rubber into cement-clay composites and found that it could significantly improve the deformability and energy absorption characteristics of the material; when the rubber content is appropriately controlled, ductility can be improved without compromising the fundamental load-bearing capacity. Bayrak et al. [14] introduced tire char as a partial aggregate replacement into geopolymer concrete and systematically investigated the evolution of its mechanical properties; the results showed that the synergistic effect of the porous structure of tire char and the rubber component could improve the crack resistance of concrete, but led to a reduction in elastic modulus. Zhu et al. [6] analyzed the influence of rubber particles on the fracture properties of self-compacting concrete and revealed the microscopic mechanism by which rubber particles improve the crack resistance of concrete by inhibiting crack propagation and absorbing fracture energy. Fan et al. [15] incorporated waste tire rubber particles into granular fillings, which could effectively reduce the lateral expansion force of soil and buffer the expansion stress of soil by utilizing the elastic deformation capacity of rubber particles. The study by Al-Subari and Ekinci [16] indicated that the incorporation of crumb tire rubber could optimize the stress-strain relationship of artificially cemented alluvial soil, but its compressive strength exhibited a phased decrease with the increase in rubber content, requiring mix proportion adjustment to balance the requirements of strength and toughness. Yadav and Tiwari’s [17] study found that the incorporation of crumb rubber reduces the strength of cement-stabilized clayey soil, but effectively improves its brittle failure characteristics and transforms its behavior from brittle to ductile.

Previous studies indicate that the addition of rubber particles improves the deformability and energy absorption characteristics of the material, but its soft nature tends to reduce the mechanical properties of concrete. This behavior is primarily attributed to the elasticity and hydrophobic nature of rubber particles, resulting in weak interfacial bonding with the cement paste [18,19]. Moreover, the incorporation of rubber alters the internal pore structure of concrete, manifested as an increase in air content and porosity, which ultimately exerts an adverse effect on the mechanical properties and durability of the material [20]. Therefore, in the preparation of rubberized concrete, optimizing the rubber content to minimize performance degradation is a critical prerequisite for balancing resource utilization and material performance. Particular attention should be paid to investigating the replacement ratio of cement in concrete, as cement production has become a major source of greenhouse gas emissions, mainly due to the carbon dioxide (CO_2_) released during the cement manufacturing process [21,22]. Accordingly, identifying an appropriate cement replacement content can reduce carbon emissions and minimize the degradation of concrete performance to the greatest extent.

Several scholars have recognized rubber particle size as a key factor affecting the mechanical properties of rubberized concrete and conducted corresponding studies. Zhao et al. [23] replaced fine aggregate with coarse and fine rubber particles to investigate the effect of rubber particle size on the mechanical properties of concrete. The results showed that concrete with fine rubber particles exhibited superior strength, more uniform stress distribution, and greater microstructural integrity, which was attributed to its higher packing density, lower porosity, and more uniform shrinkage behavior. Qureshi et al. [24] replaced natural sand in concrete with waste tire rubber aggregate and examined the effect of different rubber particle sizes on the mechanical properties of rubberized concrete. The results indicated that finer rubber particles provided excellent cohesion and fewer voids, leading to a significant increase in compressive strength compared with medium and coarse particles. Abbas et al. [25] tested the mechanical properties of concrete specimens with different rubber particle sizes and found that fine particles (<1 mm) resulted in high porosity, coarse particles (7–10 mm) caused a more significant strength reduction, and medium particles (3–5 mm) yielded the optimal strength performance. Du et al. [26] studied concrete with six rubber particle sizes (0.1 mm, 0.5 mm, 2 mm, 5 mm, 10 mm, and 20 mm) using split Hopkinson pressure bar (SHPB) tests to record dynamic compressive properties. The results demonstrated that rubberized concrete with particle sizes larger than 5 mm exhibited lower dynamic strength. It can be concluded from the studies on rubber particle size and content that optimizing rubber particle size and content is essential to achieve a balance between strength and flexibility in rubberized concrete.

Studies on rubberized concrete provide an important insight: rubber particles can be introduced as filling aggregates into cemented tailings backfill. As a technology for disposing mine solid waste, cemented tailings backfill technology mixes tailings with cementitious materials to backfill mined-out areas [27,28,29,30,31]. The backfill technology realizes the utilization of solid waste, reduces the risk of surface subsidence, and maintains the stability of the stope [32,33,34]. As the loading duration of cemented tailings backfill in the stope increases, the backfill tends to exhibit brittle characteristics, accompanied by block spalling and dilution problems [35,36,37].

In view of the aforementioned studies on concrete incorporating rubber particles, it is expected that adding rubber particles with elastic deformability and flexibility into cemented tailings backfill can alleviate the brittle failure of the backfill. Therefore, observing and investigating the crack initiation and propagation behavior of rubber particle-modified cemented tailings backfill is of great significance. Digital image correlation (DIC) is a non-contact optical measurement method with simple operation, which can obtain full-field strain distribution and characterize the cracking behavior of backfill [38,39,40]. Applying DIC technology during the failure process of backfill enables the exploration of fracture evolution and the clarification of failure mechanisms [41,42].

Existing research on rubber modification has mainly focused on conventional concrete systems, whereas investigations on rubber-modified cemented tailings backfill remain limited. More importantly, current studies rarely address the following key issues: the coupled effect of rubber particle size and content on the strength–ductility balance of CTB; the full-field strain evolution and crack propagation characteristics during failure; the energy conversion and dissipation mechanism of rubber-modified CTB; and the microstructural origin of mechanical degradation and ductility enhancement.

Furthermore, in the present mix design, rubber particles replace part of the cement. Therefore, mechanical responses are governed by a dual mechanism: intrinsic low elastic modulus and elasticity of rubber, and reduction of cementitious hydration products. Clarifying this coupled mechanism is essential for understanding the performance evolution of rubber–cemented tailings backfill (R-CTB). To address these gaps, this study establishes a control group (0% rubber) to quantitatively evaluate the modification effect of rubber on CTB and reveals the effects of rubber particle size (20–80-mesh) and content (2–8%) on mechanical properties, strain field and energy dissipation of R-CTB. The scope covers mechanical tests at 7 d and 28 d curing ages, DIC full-field strain measurement, energy dissipation calculation, and SEM micro-morphology and fractal dimension analysis.

## 2. Materials and Methods

### 2.1. Experimental Materials

The experimental raw materials were purchased in Shandong and Sichuan Province, China, with the detailed specifications and sources as follows:

(1) Mine tailings (WS)

**Figure 1:**
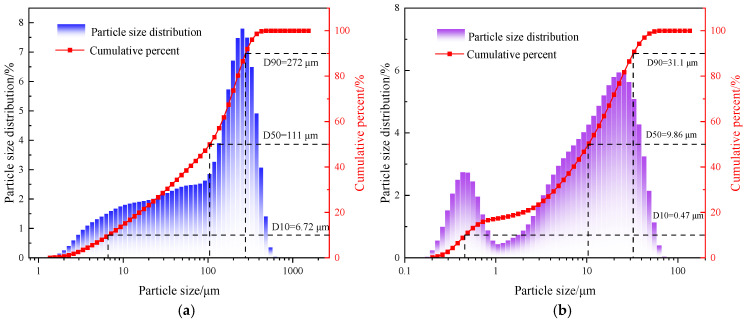
Particle size distribution of raw materials: (**a**) tailings; (**b**) cement.

**Figure 2:**
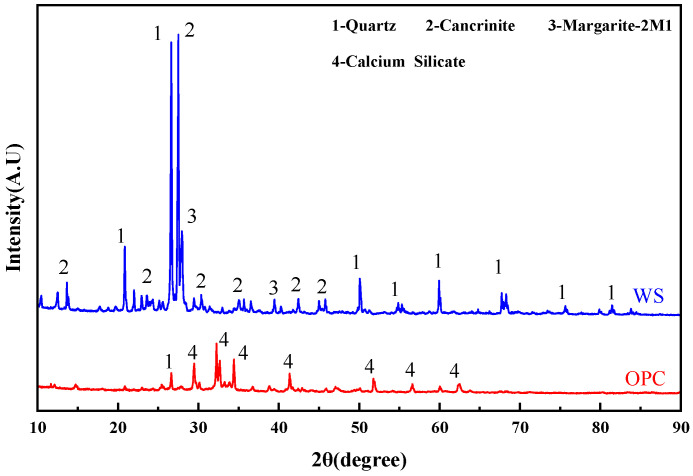
XRD analysis of raw materials.

Served as the main aggregate of the cemented tailings backfill. Particle size distribution was characterized using a Malvern 3000 laser analyzer, as presented in Figure 1. The median particle size (D50) of the tailings employed in the experiment was 111 μm (0.111 mm). X-ray diffraction (XRD) pat-terns were used to analyze the mineral phases of the tailings, as shown in Figure 2. The tailings were mainly composed of low-activity silica (quartz). The chemical composition of the tailings was determined by X-ray fluorescence (XRF) spectrometry, as listed in Table 1.

(2) Ordinary Portland cement (OPC)

Grade 42.5, the cementitious material of the backfill. A Malvern 3000 laser particle size analyzer was used to characterize the particle size distribution of the cement, as presented in Figure 1. XRD patterns were used to analyze the phases of the cement, as shown in Figure 2. The cement primarily contained crystalline phases such as quartz, calcium silicate. The chemical composition of the cement was determined by X-ray diffraction patterns, as listed in Table 2.

(3) Waste tire rubber particles

Rubber particles were supplied by Huayi Rubber Co., Ltd. of Chengdu, China. And produced from recycled waste tires through standardized processing. Steel wires were removed, and the tire matrix was crushed and screened to obtain graded particles of 20-, 40-, 60-, and 80-mesh (0.85, 0.425, 0.25, and 0.18 mm). Cleaning and drying were applied to remove contaminants and moisture, while textile and non-rubber residues were eliminated during crushing and screening. No obvious metal or textile impurities were observed in the final particles.

### 2.2. Mix Proportion Design

In this study, mechanical performance tests of the backfill were designed. To satisfy the requirements of backfill strength, fluidity, and cost, the experimental mix proportions are presented in Table 3. The cement–tailings ratio of the backfill specimens was 1:4, with a mass concentration of 70%, and the water-to-cement ratio was 7:15. Four levels of rubber particle content were set as internal replacements for cement [16], accounting for 2%, 4%, 6%, and 8% of the specimen mass, respectively. Four groups of rubber particle sizes were used [26], in the order of 80-, 60-, 40-, and 20-mesh. The experiment was carried out in batches with curing ages of 7 d and 28 d, with 16 groups of specimens per batch. The main objective was to investigate the general early-age, medium- and long-term mechanical properties of the R-CTB.

It should be clearly noted that in the present study, rubber particles were introduced as an internal replacement for cement. Therefore, increasing rubber content simultaneously leads to a reduction in cement content. The mechanical response observed in R-CTB specimens is thus the combined effect of rubber incorporation and cement reduction.

### 2.3. Specimen Preparation

To investigate the performance of backfill modified with rubber particles, cement was replaced by rubber particles. The detailed preparation procedure is as follows:

First, tailings, cement, mixing water, and rubber particles were accurately weighed. Then, the tailings and cement were placed into a mixer and dry-mixed in accordance with the designed proportions. Subsequently, the rubber particles were added at the designed content and mixed until fully dispersed. After uniform dry mixing, the designed amount of water was introduced, and the mixture was stirred thoroughly to obtain a homogeneous slurry. The prepared slurry was cast into cubic molds with dimensions of 70 mm × 70 mm × 70 mm. After removing air bubbles via a vibrating table, the molds were placed in a standard constant temperature and humidity curing box (TYC-HTX) and left to stand for 24 h at 20 ± 1 °C and 95% relative humidity for initial setting before demolding. All specimens were clearly labeled, then transferred to standard constant temperature and humidity curing box for curing until the target ages of 7 d and 28 d. The curing conditions were set as follows: temperature of 20 °C and relative humidity of 95%.

### 2.4. Test Methods

**Figure 3:**
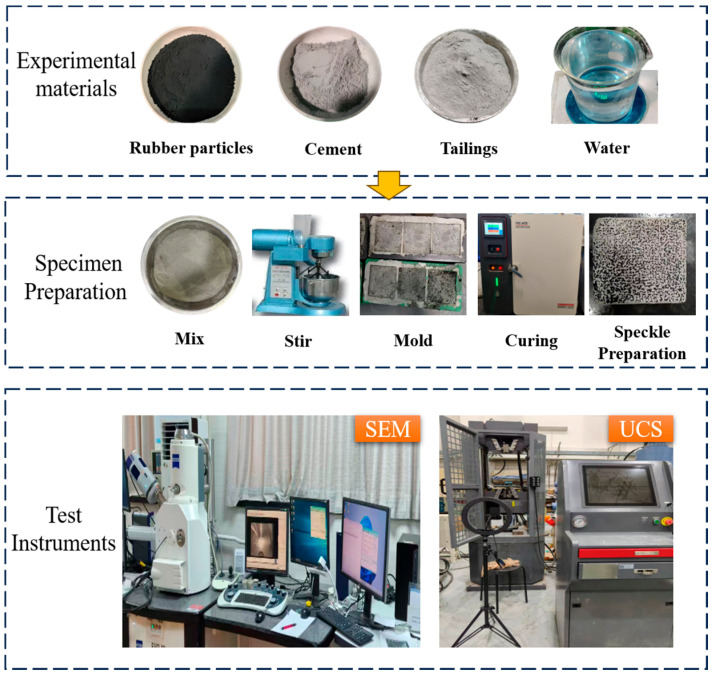
Test flow chart.

After reaching the specified curing ages, a series of tests were performed on the specimens, including uniaxial compressive strength (UCS) testing, digital image correlation (DIC) measurement, and scanning electron microscopy (SEM) observation. The test flow is illustrated in Figure 3.

(1) Uniaxial Compressive Strength (UCS) Test

A WAW-300 universal testing machine (from Zhejiang Chenxin Machine Equipment Co., Ltd., Shaoxing, China) was used to conduct uniaxial compression tests on the backfill specimens at the target curing ages. Displacement-controlled loading was adopted at a constant rate of 0.5 mm/min. During loading, the computer system recorded the displacement and load of the testing machine in real time. After completion of loading, the specimen dimensional parameters were input into the system, which automatically calculated the stress and strain values throughout the loading process, and simultaneously recorded key parameters such as the peak stress and peak strain of the specimen. After the test, the uniaxial compressive strength, elastic modulus, and other mechanical indicators were derived.

(2) Digital Image Correlation (DIC) Test

Artificial speckles were prepared on the surface of 28 d-cured backfill specimens by first spraying a uniform white matte base coat, and black spots were applied to the surface of the specimens using a specialized roller. Specimens were fixed on the universal testing machine with a rigid fixture to prevent rigid body displacement during the test.

The self-assembled non-contact DIC system consisted of an imaging-recording unit, a lighting unit, a synchronous control unit and a data processing unit, with detailed settings as follows:

Imaging unit: An OPPO Find X8 smartphone (50 MP main camera, 1/1.43 inch sensor, 23 mm focal length) was fixed at the center of a ring light source on a height-adjustable metal optical bracket. Its lens was perpendicular to the specimen surface (600 mm horizontal distance), and set to 1080 P video recording mode. Video recording was triggered synchronously with the linear displacement-controlled loading to ensure time consistency of load and deformation data.

Lighting unit: An 18 W dimmable ring light source (5500 K, ≥90% uniform illumination) was fixed on the bracket, with the smartphone coaxially arranged at its hollow center. Its light-emitting surface was parallel to the specimen surface, providing shadowless diffuse illumination and avoiding speckle overexposure/underexposure.

After the test, the video was imported into Vic-2D for frame-by-frame analysis, and the frame rate is 25.

(3) Scanning Electron Microscopy (SEM) Test

A GeminiSEM 500 scanning electron microscope was employed. Five representative fragments from mechanical tests were selected and cut into thin slices with a thickness of 5 mm. After copper mounting and carbon coating, the slices were dried continuously in an oven at 60 °C for 12 h. Following vacuum extraction, the microstructure of the specimens was observed at a magnification of 2000×.

## 3. Results and Discussion

### 3.1. Mechanical Properties of R-CTB

#### 3.1.1. Uniaxial Compressive Strength

**Figure 4:**
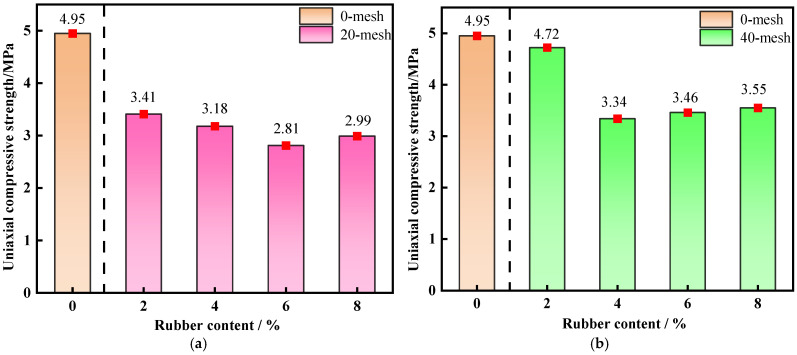

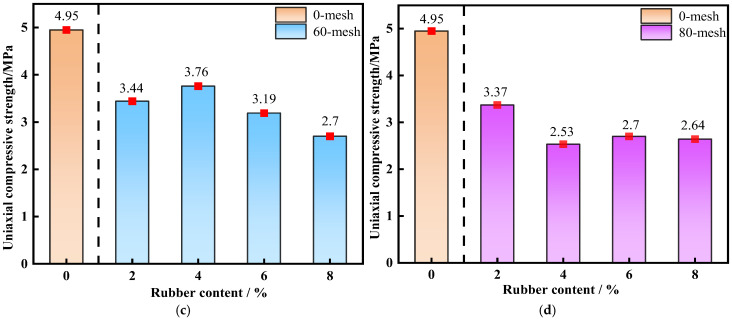
Variation trend of compressive strength of R-CTB with different particle sizes compared with the control group at 28 d curing age: (**a**) 20-mesh; (**b**) 40-mesh; (**c**) 60-mesh; (**d**) 80-mesh.

Figure 4 presents the variation in the uniaxial compressive strength (UCS) of backfill specimens with rubber content at a curing age of 28 d, in direct comparison with the 0% rubber control group (traditional CTB without rubber addition).

By introducing the control group, the absolute strength reduction caused by rubber incorporation and cement reduction can be quantitatively evaluated. All R-CTB groups show lower UCS than the control group, confirming that cement replacement by rubber inevitably weakens the bearing capacity.

It can be observed from Figure 4 that the UCS of backfill containing 20-mesh, 40-mesh, and 80-mesh rubber particles first decreases and then increases with rising rubber content. In contrast, the strength of backfill with 60-mesh rubber particles displays a trend of increasing first and then decreasing.

The 40-mesh R-CTB exhibits the highest strength. At a rubber content of 2%, the compressive strength of R-CTB reaches 4.72 MPa, the maximum value among all particle size and content combinations. As the content further increases to 4%, the strength decreases by 29.23%. At 6% and 8% contents, the strength rises slowly but insignificantly, indicating that the increase in rubber content has only a minor effect on strength in this range.

For 20-mesh R-CTB, the strength decreases by 6.74% at 4% content compared with 2% content, and by 17.59% at 6% content relative to 2% content. When the content reaches 8%, the strength recovers slightly by only 0.18 MPa.

The strength of 60-mesh and 80-mesh R-CTB decreases noticeably overall. For 60-mesh R-CTB at low contents, the strength increases marginally by just 9.3% with rising rubber content, followed by a rapid decline at higher contents. At 8% content, the strength falls to 2.7 MPa, 2 MPa lower than the peak value, representing a substantial reduction.

The strength evolution of 80-mesh R-CTB is similar to that of 40-mesh R-CTB: the strength drops sharply by 24.92% as the content increases from 2% to 4%, and changes only slightly with further increases in content, remaining at a low level overall. At 8% content, the strength is 2.64 MPa. In general, the strength gap between R-CTB and traditional CTB expands with increasing rubber content and decreasing particle size; combinations of higher mesh number + higher rubber content yield the lowest uniaxial compressive strength.

**Figure 5:**
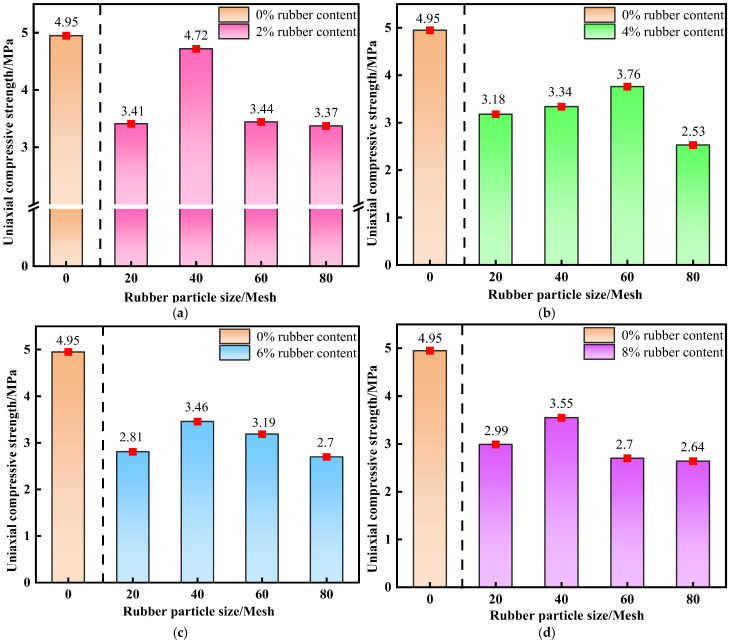
Variation trend of compressive strength of R-CTB with different rubber contents compared with the control group at 28 d curing age: (**a**) 2% rubber content; (**b**) 4% rubber content; (**c**) 6% rubber content; (**d**) 8% rubber content.

Figure 5 shows the variation of uniaxial compressive strength of backfill with decreasing rubber particle size at a curing age of 28 d, together with the 0% rubber control group (traditional CTB) as the baseline for quantitative comparison. The strength of R-CTB for each content rises first and then falls with the reduction of particle size. When particle size decreases from 20-mesh to 40-mesh, the strength approaches the control group gradually; as particle size further reduces to 60-mesh and 80-mesh, the strength moves away from the control group and decreases significantly.

When the particle size decreases from 20-mesh to 40-mesh, the strength increases by 1.31 MPa, 0.16 MPa, 0.65 MPa and 0.56 MPa, with growth rates of 38.41%, 5.03%, 23.13% and 18.72% respectively. The strength drops rapidly as the particle size continues to decrease below 40-mesh. Rubber content regulates the effect of particle size on strength, and the strength of 80-mesh R-CTB is almost equal to that of 20-mesh.

Among the four contents, only 40-mesh R-CTB shows a significant strength increase at low or high contents, while the strength changes little at other particle sizes. For moderate contents (4% and 6%), the strength remains high at 40-mesh and 60-mesh.

A 40-mesh size is the optimal particle size, and the strength peaks at 4.72 MPa with 2% rubber content, after which the strengthening effect weakens when the content exceeds 4%. Excessively fine particles (60-mesh, 80-mesh) or high contents (6%, 8%) reduce the strength of R-CTB. The combination of 40-mesh and 2% rubber content presents the best mechanical performance, but excessively fine particles (60-mesh, 80-mesh) or high contents (6%, 8%) reduce the strength of R-CTB and increase the difference from the control group.

Cement replacement directly decreases the amount of C–S–H gel and other hydration products, which weakens the load-bearing skeleton of the backfill matrix. This reduction in effective binder phase significantly lowers the compressive strength. And due to the density difference between rubber (low density) and cement, segregation and stratification occur during casting, increasing internal porosity. The entrapped air and weakly bonded interfaces act as stress concentrators under loading. The hydrophobic nature of rubber particles limits chemical bonding with hydration products, resulting in weak matrix–rubber interfacial zones. Cracks preferentially initiate and propagate along these interfaces, accelerating failure.

#### 3.1.2. Elastic Modulus

**Figure 6:**
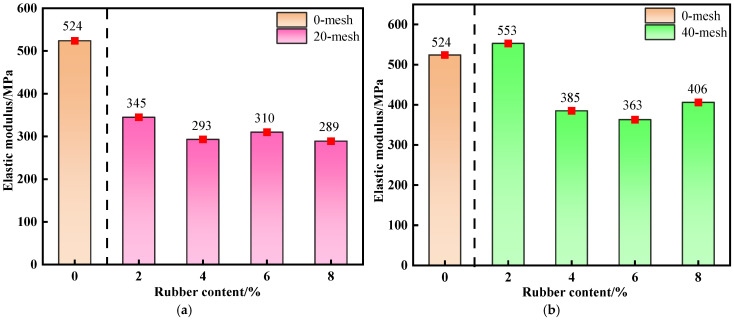

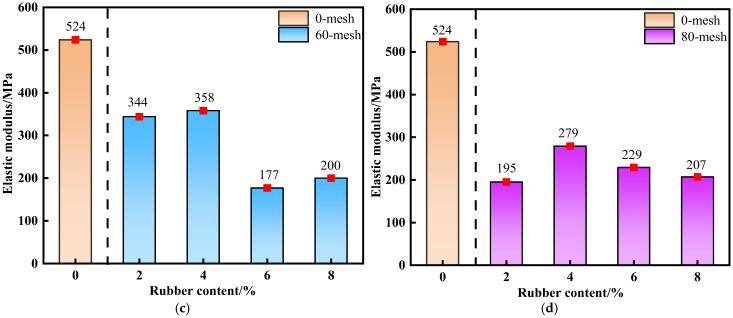
Variation trend of elastic modulus of R-CTB with different particle sizes compared with the control group at 28 d curing age: (**a**) 20-mesh; (**b**) 40-mesh; (**c**) 60-mesh; (**d**) 80-mesh.

Figure 6 presents the variation in the elastic modulus of R-CTB with increasing rubber content at a curing age of 28 d, together with the elastic modulus of the 0% rubber control group for quantitative comparison. Elastic modulus quantifies a material’s resistance to deformation in the elastic deformation stage. For rubber particle-modified backfill, its evolution directly reflects the influence of rubber particles on the elastic modulus of the backfill [43].

The control group shows the highest elastic modulus, serving as the baseline for elastic modulus evaluation. All R-CTB groups have lower elastic modulus than traditional CTB, which is jointly caused by the low modulus of rubber and the reduction of cement hydration products.

As shown in Figure 6a–d, the elastic modulus of R-CTB first increases and then decreases with the reduction of particle size, and the difference from the control group becomes more significant at higher rubber contents. The 40-mesh with 2% rubber content maintains the highest elastic modulus closest to the control group, while the 80-mesh with 8% content shows the largest elastic modulus loss.

By comparing with the control group, it can be distinguished that the elastic modulus reduction of R-CTB includes two parts: the inherent softening effect of rubber elastic phase, and the strength loss caused by insufficient cement hydration.

**Figure 7:**
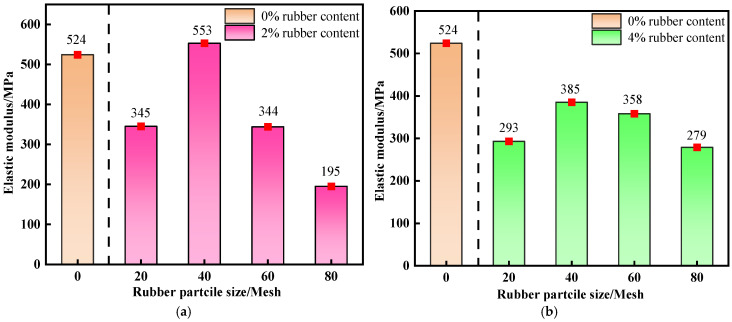

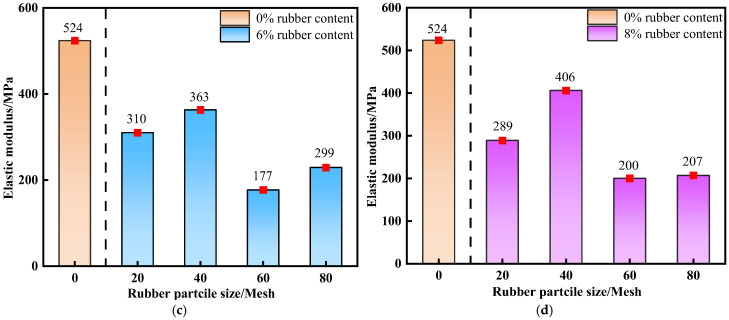
Variation trend of elastic modulus of R-CTB with different rubber contents compared with the control group at 28 d curing age: (**a**) 2% rubber content; (**b**) 4% rubber content; (**c**) 6% rubber content; (**d**) 8% rubber content.

Figure 7 presents the variation in the elastic modulus of R-CTB with decreasing rubber particle size at a curing age of 28 d, together with the 0% rubber control group (traditional CTB) as the elastic modulus baseline for quantitative comparison. The elastic modulus of R-CTB at each content exhibits a trend of increasing first and then decreasing with the reduction of particle size. Although the two content groups display the same variation trend, their change amplitudes differ significantly. When the particle size decreases to 40-mesh, the elastic modulus of R-CTB with 2% rubber content increases by 208 MPa, with a growth rate of 60.29%, while that of R-CTB with 4% rubber content increases by 92 MPa, with a growth rate of 31.39%. When the particle size further decreases to 60-mesh, the elastic modulus of R-CTB with 2% rubber content drops rapidly to the original level, whereas that of R-CTB with 4% rubber content only decreases by 27 MPa. This indicates that rubber content affects the variation amplitude of particle size on the elastic modulus of R-CTB.

As shown in Figure 7c, the elastic modulus of R-CTB remains high and basically constant at relatively large particle sizes. With a further reduction in particle size, the elastic modulus of R-CTB decreases sharply by 187 MPa, representing a reduction of 51.37%, at which point particle size exerts a highly significant effect on the reduction of elastic modulus.

As shown in Figure 7d, the elastic modulus of R-CTB reaches the maximum at 40-mesh, and decreases rapidly to a sustained low level with further reduction in particle size.

At a curing age of 28 d, the combination of 40-mesh particle size and 2% rubber content exhibits the optimal elastic modulus, and the influence of content variation on the elastic modulus is relatively mild under this combination. The overall elastic modulus of 20-mesh particle size is low but fluctuates slightly with content. In contrast, when excessively fine particle sizes (such as 60-mesh and 80-mesh) are coupled with high contents (such as 6% and 8%), the elastic modulus attenuates significantly. In particular, the combination of 80-mesh particle size and 8% rubber content maintains a persistently low elastic modulus, representing the combination with the poorest elastic modulus.

In addition, rubber content alters the influence amplitude of particle size on the elastic modulus. At low contents, the particle size effect is more prominent, and the difference in elastic modulus is mainly determined by particle size; at high contents, the variation in elastic modulus is dominated by rubber content.

The elastic modulus is particularly sensitive to binder content. Since rubber replacement reduces cement dosage, the elastic modulus reduction is not merely caused by the low modulus of rubber itself, and the modulus attenuation should be understood as a combined consequence of matrix softening and the intrinsic low elastic modulus of rubber particles.

#### 3.1.3. Peak Strain

**Figure 8:**
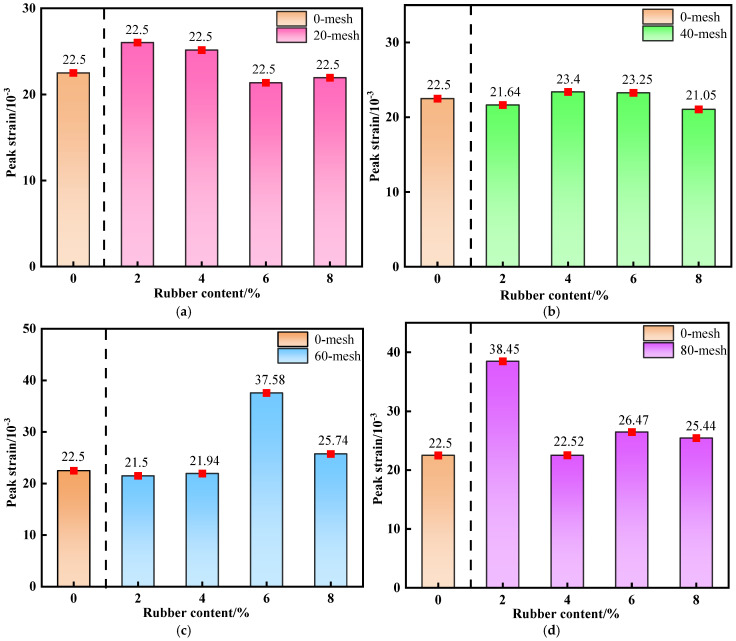
Variation trend of peak strain of R-CTB with different particle sizes compared with the control group at 28 d curing age: (**a**) 20-mesh; (**b**) 40-mesh; (**c**) 60-mesh; (**d**) 80-mesh.

Peak strain reflects the maximum deformation of cemented backfill prior to reaching its ultimate bearing capacity, serving as a key indicator characterizing the material’s ductility and deformation compatibility. Compared with the brittle control group (traditional CTB), all R-CTB groups show higher peak strain, confirming the ductility enhancement brought by rubber. As shown in Figure 8a–d, at moderate particle sizes (i.e., 40-mesh and 60-mesh), the peak strain of R-CTB exhibits a gradual increasing trend with rising rubber content, and the strain increment relative to the control group increases accordingly, indicating that the incorporation of rubber particles at these sizes can enhance the ductility of the backfill.

The control group has the smallest peak strain and typical brittle failure. With the increase of rubber content and the decrease of particle size, the gap in peak strain between R-CTB and the control group gradually increases, which directly proves that rubber can improve the ductility of CTB.

Larger rubber particles exert a limited effect on improving deformability at low contents. Although the peak strain of R-CTB at 80-mesh shows a decreasing trend with increasing rubber content, the specimens still maintain relatively high peak strain at 6% and 8% contents.

**Figure 9:**
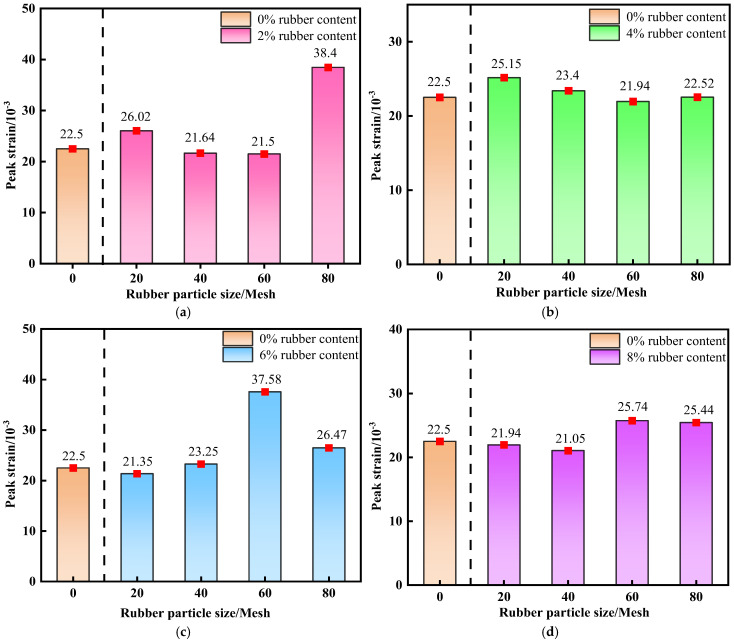
Variation trend of peak strain of R-CTB with different rubber contents compared with the control group at 28 d curing age: (**a**) 2% rubber content; (**b**) 4% rubber content; (**c**) 6% rubber content; (**d**) 8% rubber content.

As shown in Figure 9a–d, overall, at the same rubber content, the smaller the particle size of R-CTB, the higher the peak strain, together with the 0% rubber control group (traditional CTB) as the ductility baseline for quantitative comparison.

All R-CTB groups exhibit higher peak strain than the control group, directly confirming that rubber particles significantly improve the ductility and deformation capacity of cemented tailings backfill.

At 2% and 4% contents, the peak strain tends to decrease first and then increase, whereas at 6% and 8% contents, relatively large peak strains occur at finer particle sizes (i.e., 60-mesh and 80-mesh).

With increasing rubber content, the peak strain shows an overall upward trend, and the ductility improvement is most pronounced at moderate particle sizes (40-mesh and 60-mesh). This indicates that rubber particles within this size range can participate more effectively in the deformation compatibility of the matrix and delay the failure process. In comparison, larger rubber particles have a limited effect on improving peak strain at low contents, yet can still maintain relatively high deformability at high contents.

#### 3.1.4. Comparison of Mechanical Properties of R-CTB Under Early-Age and Medium-to-Long-Age Curing Conditions

**Figure 10:**
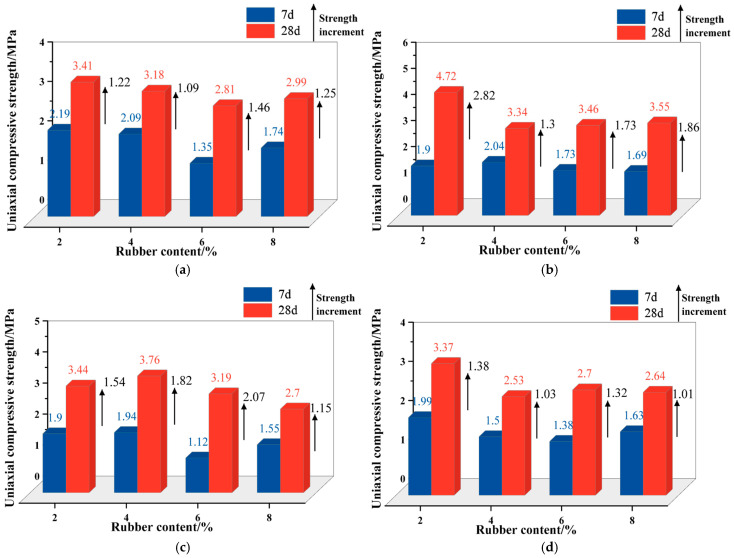
Comparison of mechanical properties of R-CTB at 7 d and 28 d curing ages: (**a**) 20-mesh; (**b**) 40-mesh; (**c**) 60-mesh; (**d**) 80-mesh.

Figure 10a presents a comparison of the mechanical properties of 20-mesh R-CTB with different rubber contents at curing ages of 7 d and 28 d. For 7 d cured R-CTB, the peak strength reaches the maximum at 2% rubber content and decreases gradually with increasing content. For 28 d cured R-CTB, the peak strength is also the highest at 2% rubber content, showing the same trend with content as the 7 d specimens. From 7 d to 28 d, the peak strength of 20-mesh R-CTB increases by 1.22 MPa, 1.09 MPa, 1.46 MPa and 1.25 MPa successively with rising rubber content. Although the strength increment is the largest at 6% content, its 28 d peak strength remains the lowest among all groups.

Figure 10b compares the mechanical properties of 40-mesh R-CTB with different contents at 7 d and 28 d. At 7 d, the peak strength peaks at 4% rubber content and bottoms out at 8% content. At 28 d, the peak strength becomes the highest at 2% content, far exceeding other groups, while the strength of other contents stabilizes at approximately 3.4 MPa. The strength increments from 7 d to 28 d are 2.82 MPa, 1.30 MPa, 1.73 MPa and 1.86 MPa with increasing content, with the 2% content group showing the largest gain and the final highest peak strength. Notably, at 7 d, the strength of all content groups varies slightly and centers around 1.8 MPa. At 28 d, only the 2% content group exhibits a pronounced strength improvement, with negligible differences among the other groups.

Figure 10c shows the mechanical performance comparison of 60-mesh R-CTB at 7 d and 28 d. At 7 d, the peak strength is maximized at 4% rubber content and minimized at 8% content. At 28 d, the peak strength still peaks at 4% content, with a trend nearly consistent with that at 7 d. The strength increments from 7 d to 28 d are 1.54 MPa, 1.82 MPa, 2.07 MPa and 1.05 MPa as content increases. The 6% content group records the largest strength gain, and its 28-d strength is notably higher than that of the 6% content group in Figure 10a (which shows a similar trend). Nevertheless, low-content R-CTB still presents higher overall strength and larger growth amplitudes.

Figure 10d compares the mechanical properties of 80-mesh R-CTB at 7 d and 28 d. At 7 d, the peak strength is the highest at 2% rubber content; unlike previous cases, the 4% content group shows relatively low strength. At 28 d, the 2% content group remains the strongest, with a value significantly higher than the rest, and the 4% content group shows the lowest strength. The strength increments from 7 d to 28 d are 1.38 MPa, 1.03 MPa, 1.32 MPa and 1.01 MPa with increasing content. Under the 80-mesh condition, the strength gains of R-CTB at 28 d are relatively modest.

R-CTB at both 7 d and 28 d generally achieves the highest peak strength at low rubber contents, and the trend of peak strength versus content is largely consistent between the two curing ages. Obvious strength enhancements occur at 40-mesh and 60-mesh, and prolonged curing also boosts the strength of high-content R-CTB noticeably. Low rubber contents exert minor negative effects on the peak strength of backfill, whereas excessively high contents reduce the overall load-bearing capacity because rubber particles do not participate in the hydration reaction. Extending the curing period from 7 d to 28 d promotes more complete hydration of cementitious materials and a denser microstructure, leading to higher overall strength. High-content R-CTB suffers from inherently low early-age strength due to the large volume of rubber, and the curing age extension cannot reverse the intrinsic strength weakening caused by high rubber contents.

Incorporating rubber particles reduces the compressive strength and elastic modulus of backfill, with the reduction increasing with rubber content, while improving ductility that strengthens with higher content. This conclusion is consistent with [6,12,13,14,16,17], which all show that rubber particles, though reducing cement-based concrete strength, improve brittleness and realize the brittleness-to-ductility transformation.

The influence of rubber particle size on backfill mechanical properties differs from existing studies, which can be explained by test parameter differences. Zhao et al. [23]. showed that coarse particles (size > 1 mm) reduce mechanical properties while fine particles improve them; their conclusion on 0.3 mm fine particles is consistent with this study’s optimal 40-mesh (≈0.425 mm) particle size, and particle shape differences may affect strength. Qureshi et al. [24] studied particles > 2 mm and found strength decreases with size; this study focuses on particles < 1 mm, with little size impact presumably due to the narrow size range. Du et al. [26] found significantly lower dynamic strength for particles > 5 mm, and this study complements the above research to improve the related system.

### 3.2. Strain Characteristics During R-CTB Failure

Digital image correlation (DIC) technique enables non-contact measurement of surface strain on the backfill during deformation, and directly visualizes the deformation state of R-CTB under axial stress.

**Figure 11:**
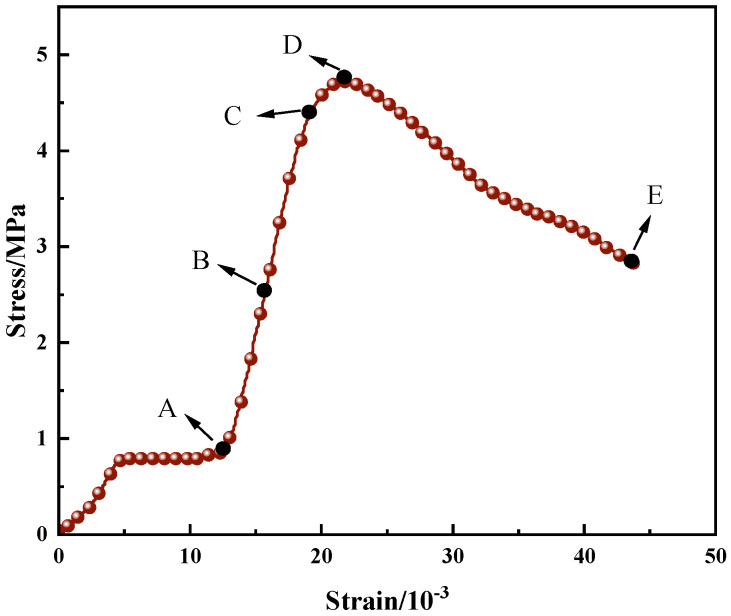
Schematic diagram of selected representative loading points.

Five representative loading points are selected on the stress-strain curve, as illustrated in Figure 11. Point A denotes the onset of the linear elastic stage, Point B the midpoint of the linear elastic stage, Point C the yield point, Point D the peak load point, and Point E the post-peak failure point. Point E is determined by the compression termination command set in the testing machine. VIC-2D 7.0 software is adopted to analyze the axial strain digital speckle images of the R-CTB at the five loading points. During the DIC analysis with VIC-2D software, the unit length is calibrated, the number of meshes is set to 49 to ensure that at least three speckles are contained in each mesh, and the mesh step is set to 11.

All strain data in this study, including the stress-strain curve in Figure 11, are defined as axial engineering strain with the unit of percentage (%).

**Figure 12:**
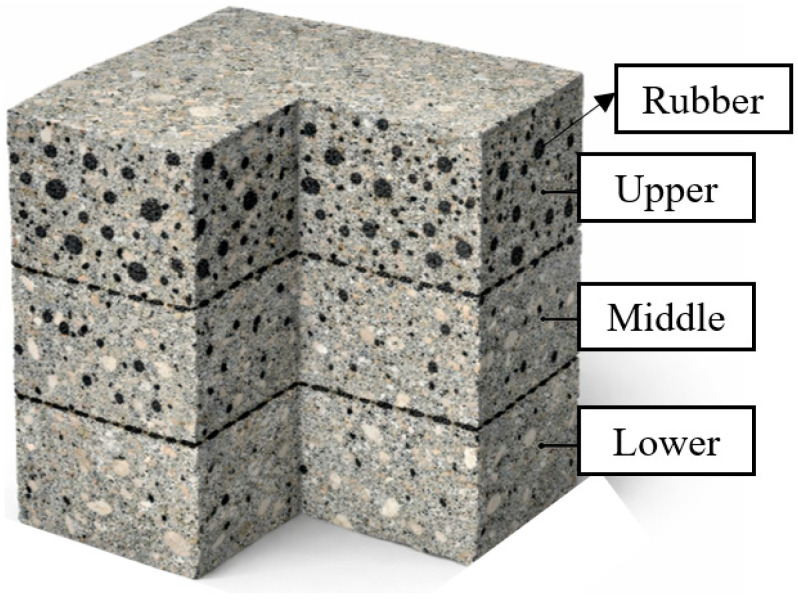
Schematic diagram of zone division.

The low density of waste tire rubber particles leads to their uneven distribution in the cemented tailings backfill matrix, resulting in obvious segregation and stratification phenomena in the prepared R-CTB specimens [44]. And a stratification phenomenon was also observed after the sample was properly cured. To further analyze the evolution of stratification in R-CTB with 2% rubber content and various particle sizes, local strain measurement zones were defined in the upper and lower parts of the specimen within the region of interest, and the average axial strain of the specimen was recorded. As shown in Figure 12, the upper zone was placed in the upper half of the specimen to capture the strain evolution where rubber content was relatively high, while the lower zone was set in the lower half to record the strain behavior where rubber content was low, together with the average strain over the whole specimen region; in Figure 12, ε_yy_/% represents the engineering axial strain along the *y*-axis in the image coordinate system, expressed as a percentage.

**Figure 13:**
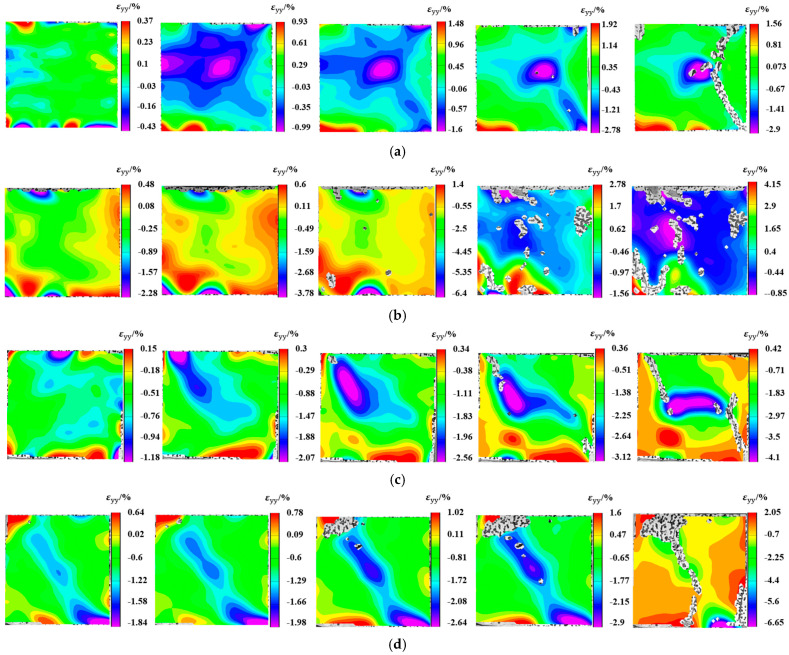
Axial strain field of R-CTB with different particle sizes at 2% rubber content: (**a**) 20-mesh; (**b**) 40-mesh; (**c**) 60-mesh; (**d**) 80-mesh.

Figure 13 presents the strain evolution of stratified cemented tailings backfill with 2% rubber content and different particle sizes. This content was chosen because the specimens exhibited overall high strength at this mixing ratio. The abscissa represents the compression time.

Figure 13 presents the evolution of the axial strain field of R-CTB with various rubber particle sizes under 2% rubber content during uniaxial compression. The negative strain values observed at crack initiation during compression of all R-CTB specimens indicate that cracks are induced by inward compressive failure. Positive strain values at crack edges signify the occurrence of outward tensile cracks.

As seen from the figure, the 40-mesh R-CTB exhibits the most uniform strain distribution. It features small strain values in the elastic stage, delayed crack initiation, scattered propagation paths, and more coordinated overall deformation, representing the group with the most stable stress transfer among all particle sizes.

The 20-mesh and 60-mesh R-CTB specimens show an inhomogeneous strain field with localized high-stress zones in the elastic stage. Cracks propagate intensively along weak interfaces, gradually coalesce with loading, and eventually lead to failure.

For the 80-mesh R-CTB, distinct through-going cracks appear at an earlier stage, accompanied by the most severe compressive failure, resulting in the lowest strength within this group.

Quantitative analysis was performed on the axial strain fields of the three zones defined in Figure 12. The upper and lower zones reflect the influence of rubber segregation on specimen deformation, while the middle zone serves as a reference.

**Figure 14:**
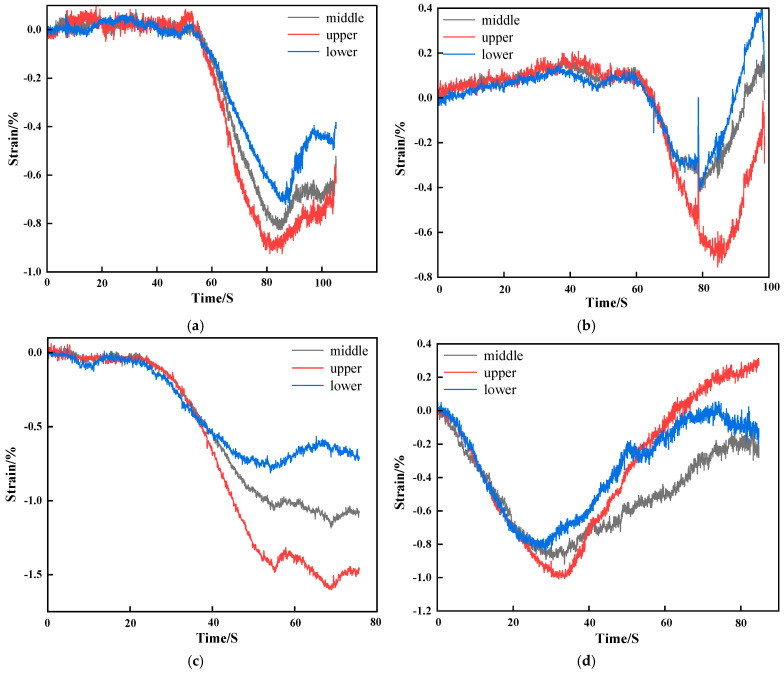
Strain evolution process of zoned R-CTB with different particle sizes at 2% rubber content: (**a**) 20-mesh; (**b**) 40-mesh; (**c**) 60-mesh; (**d**) 80-mesh.

Figure 14 presents the strain evolution curves of the three zones during uniaxial compression for specimens with different rubber particle sizes at 2% rubber content. As shown in Figure 14a–c, the axial strain curves of the three zones exhibit nearly identical trends during the compaction stage and the early elastic stage for the three particle sizes. As compression proceeds, the strain increase in the lower zone gradually slows down (the slowest growth rate), the middle zone shows a moderate growth rate, and the upper zone displays the fastest growth rate. This indicates that no significant damage occurs in the overall specimen structure during the compaction stage and early elastic stage, resulting in consistent strain trends across the three zones.

In Figure 14d, the R-CTB enters the elastic stage earlier, and the compressive strain increases progressively, which is consistent with the earlier crack initiation observed in Figure 13d. In the later stage of compression, the axial strain values of all specimens gradually decrease, and some specimens return to positive strain values, implying that partial cracks begin to open and cause axial tensile failure in the late compression phase.

As the compression test advances, the three curves diverge: the upper zone shows the fastest strain growth, and the lower zone the slowest. At the peak point, the upper zone reaches the maximum strain value, while the lower zone records the minimum. This confirms the strain discrepancy between the upper and lower parts of the specimen, indicating structural differences between the two regions. This is likely caused by the segregation and stratification of rubber. Since rubber does not participate in the hydration reaction, the upper region contains fewer hydration products responsible for load-bearing capacity than the lower region, leading to the observed strain difference.

The axial strain of 40-mesh R-CTB is the smallest, at approximately 0.4%, which aligns with the earlier conclusion that the combination of 40-mesh and 2% rubber content yields the highest strength.

### 3.3. Energy Dissipation Characteristics of R-CTB

The deformation and failure process of backfill specimens involves continuous energy exchange with the external environment [45]. During loading, the specimen continuously absorbs energy, which is then converted into releasable elastic energy and dissipated energy [46]. When the energy absorbed by the specimen exceeds its energy storage limit, part of the releasable elastic energy is released, triggering the overall failure of the specimen. According to the law of conservation of energy:

(1) $$U_{T}=U_{E}+U_{D}$$

where *U_T_* is the total energy absorbed by the specimen, *U_E_* is the releasable elastic energy stored inside the specimen, and *U_D_* is the dissipated energy of the specimen.

**Figure 15:**
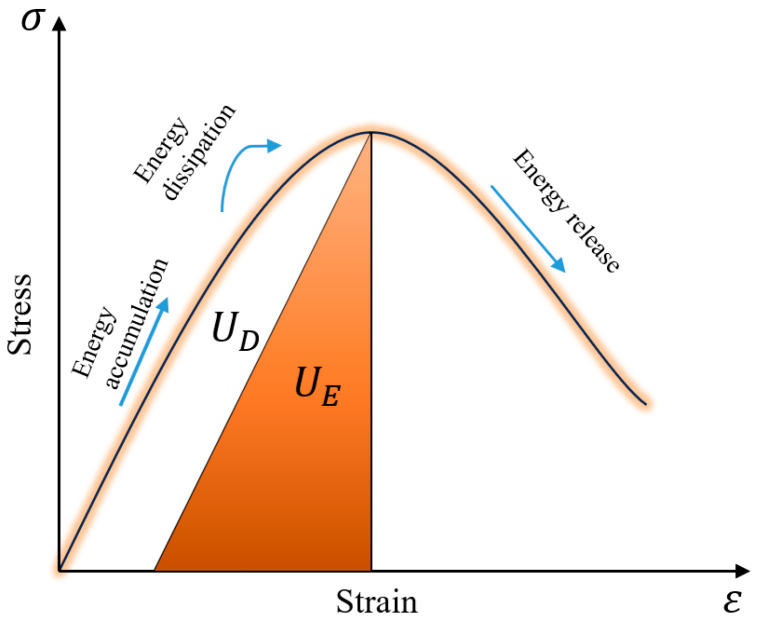
Energy evolution process of cemented backfill.

Figure 15 illustrates the internal energy evolution of the specimen. In the initial stage, the backfill absorbs energy and stores it in the form of releasable elastic energy. In the intermediate stage, the releasable elastic energy stored in the specimen exceeds its elastic limit, and part of the absorbed energy is converted into dissipated energy and released to the surroundings. In the final stage, the stored energy surpasses the specimen’s energy storage limit, leading to specimen failure and the rapid release of internally stored energy to the external environment. The energy balance equation of the specimen under uniaxial compression is expressed as

(2) $$U_{T}=\int_{0}^{\varepsilon }\sigma d\varepsilon$$

(3) $$U_{E}=\frac{\sigma^{2}}{2E_{R}}$$

where *E_R_* is the elastic modulus of the specimen.

Based on the law of conservation of energy and the energy balance equation of the specimen, combined with the stress-strain curves of 28-d cured R-CTB, the values of *U_T_*, *U_E_*, and *U_D_* at each loading point can be calculated. The calculated results are presented in Table 4.

In Figure 11, loading point C represents the damage stress point—the critical stress at which the material transitions from the elastic deformation stage to the damage evolution stage. Loading point D denotes the peak stress point.

To clarify the energy evolution laws at these two loading points in Table 4, the elastic strain energies corresponding to the damage stress point C and peak stress point D are denoted as $U_{E}^{C}$ and $U_{E}^{D}$, respectively, and the characteristic value of the total input energy at peak stress point D is labeled $U_{T}$.

The variation rates of elastic energy at the damage stress point and peak stress point are defined as k and m [47], respectively:

(4) $$k=\frac{U_{E}^{D}}{U_{T}}$$

(5) $$m=\frac{U_{E}^{C}}{U_{T}}$$

**Figure 16:**
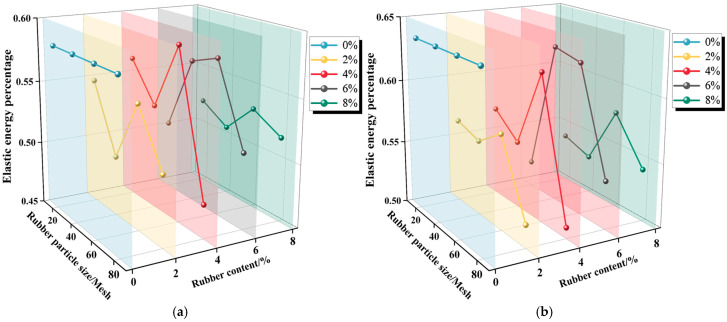
Evolution of m and k for R-CTB with different particle sizes and rubber contents: (**a**) m; (**b**) k.

Figure 16 shows the evolution of characteristic values m and k for R-CTB with different rubber particle sizes and contents at 28 d curing age, together with the 0% rubber control group (traditional CTB) as the energy storage baseline for quantitative comparison.

As shown in Figure 16a,b, both m and k first increase and then decrease with the rise in rubber particle content in R-CTB. This indicates that a higher rubber content reduces the compactness of R-CTB, leading to a lower proportion of stored elastic energy and a higher proportion of dissipated energy before reaching the damage stress and peak stress; compared with the control group, both m and k of all R-CTB groups are lower, indicating that rubber incorporation significantly reduces the elastic energy storage capacity of cemented tailings backfill.

Overall, m and k first increase and then decrease with the reduction in rubber particle size in R-CTB, suggesting that smaller rubber particles result in lower compactness and weaker elastic energy storage capacity of the composite.

Moreover, by comparing the magnitudes of m and k in Figure 16a,b, it can be seen that m > k. This reveals that once R-CTB enters the inelastic deformation stage, the proportion of elastic energy gradually declines while the proportion of dissipated energy rises. At this stage, the externally input energy is mainly consumed for internal damage evolution and structural failure. The specimen gradually transitions from a stable deformation state dominated by elastic energy storage to an unstable failure stage dominated by energy dissipation, accompanied by a significant degradation of its overall bearing capacity and structural integrity.

By introducing the control group, it can be quantitatively distinguished that the reduction of m and k is jointly affected by two factors: the decrease of cement content reduces the hydration products and energy storage space, while the rubber elastic phase promotes energy dissipation and further reduces the elastic energy storage ratio.

#### 3.3.1. Analysis of Energy Conversion Process

To characterize the conversion relationship between energy accumulation and dissipation at different loading stages, an energy conversion coefficient q is defined to quantitatively reveal this process [48]:

(6) $$q=\frac{U_{E}}{U_{D}}$$

**Figure 17:**
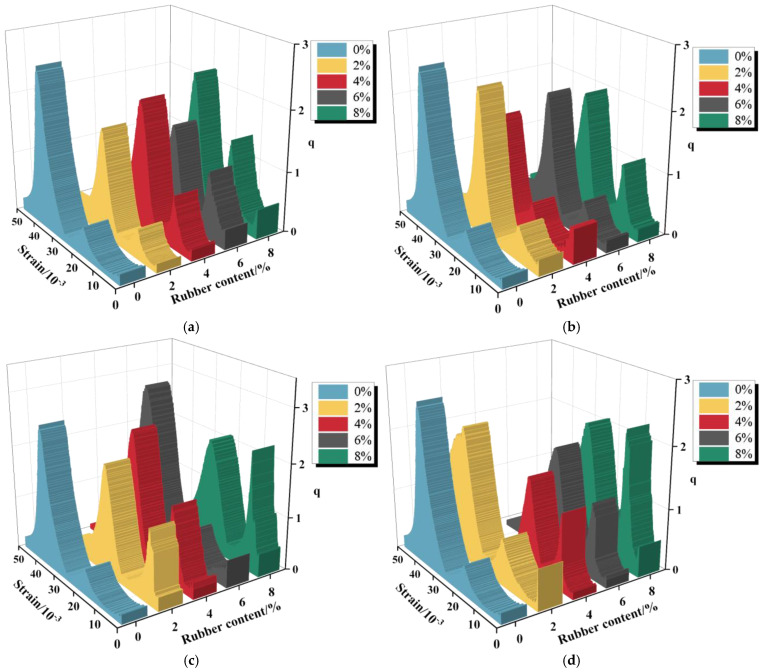
Relationship between energy conversion coefficient and strain for R-CTB with different particle sizes and rubber contents: (**a**) 20-mesh; (**b**) 40-mesh; (**c**) 60-mesh; (**d**) 80-mesh.

Figure 17 presents the evolution of the energy conversion coefficient for R-CTB with varying particle sizes and rubber contents against strain, together with the 0% rubber control group (traditional CTB) as the energy storage benchmark for quantitative comparison.

Compared with the control group, the q value of all R-CTB groups is significantly lower, indicating that rubber incorporation reduces the energy storage efficiency and increases the proportion of dissipated energy.

Finer particle sizes correspond to lower values of the energy conversion coefficient q, indicating reduced deformability and lower energy storage efficiency of R-CTB under these conditions. In general, the energy conversion coefficient decreases with increasing rubber content, suggesting that a higher rubber content lowers the energy dissipation capacity and makes the specimen more prone to accumulating the energy required for failure.

It can be observed from Figure 17 that the energy conversion coefficient exhibits a consistent trend under all test conditions. Two peaks appear in the curve; however, the first peak arises from the constant-stress loading stage (prior to the linear-elastic loading point in Figure 11) and is therefore not meaningful for analysis. Accordingly, the energy conversion process is discussed based on the second peak.

Compared with the control group, the peak value of q for R-CTB is lower, and the corresponding peak strain is higher, which demonstrates that rubber particles promote energy dissipation and delay the arrival of the peak energy storage state.

During loading, external energy input drives specimen damage. The energy conversion coefficient peaks and then declines, with the overall energy conversion still dominated by elastic energy storage. In the plastic stage, the growth rate of elastic energy slows down while dissipated energy increases. After the energy conversion coefficient reaches its maximum, the proportion of elastic energy in the specimen gradually decreases, crack propagation accelerates, and specimen fragmentation intensifies continuously until the load-bearing capacity is completely lost. By introducing the control group, it can be clearly distinguished that the reduction of the energy conversion coefficient q is the combined result of cement reduction (weakening the energy storage skeleton) and rubber addition (enhancing energy dissipation).

#### 3.3.2. Energy Dissipation Rate of Backfill

**Figure 18:**
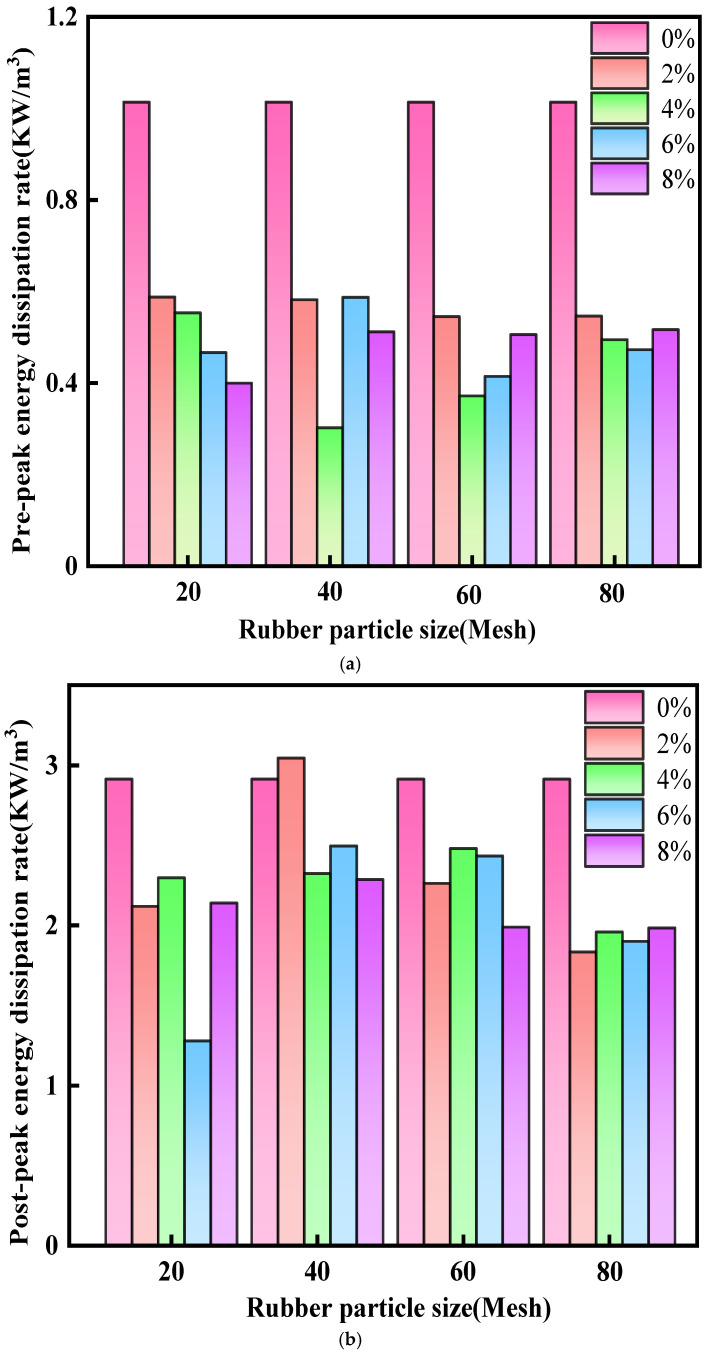
Energy dissipation rates of R-CTB with different rubber contents and particle sizes: (**a**) pre-peak energy dissipation rate; (**b**) post-peak energy dissipation rate.

The uniaxial compressive yield failure and damage of cemented tailings backfill are essentially the combined outcome of energy dissipation and energy release. The energy dissipation rates in the pre-peak and post-peak stages of cemented tailings backfill are obtained by dividing the corresponding energy consumption increments by the respective durations [49]. Figure 18 presents the pre-peak and post-peak energy dissipation rates of backfill under various test conditions, together with the 0% rubber control group (traditional CTB) as the energy dissipation baseline for quantitative comparison.

Figure 18a shows the dependence of the pre-peak energy dissipation rate of R-CTB on rubber particle size and content. A higher pre-peak rate indicates that the propagation of internal microcracks is strongly restrained before the material enters the yield stage, requiring more external energy for crack initiation, and thus corresponds to superior deformation resistance. It can be seen that the pre-peak energy dissipation rate generally increases with larger rubber particle size and lower rubber content, and the control group presents the lowest pre-peak energy dissipation rate, indicating that traditional CTB requires less energy to initiate cracks and is prone to sudden damage.

Figure 18b shows the post-peak energy dissipation rate of R-CTB as a function of curing age. The post-peak energy dissipation rate characterizes the rate of internal crack propagation and coalescence that leads to specimen failure. A higher post-peak rate implies more abrupt failure. The post-peak energy dissipation rate of R-CTB is significantly lower than that of the control group, and decreases with reducing particle size and increasing rubber content. This indicates that rubber particles can absorb energy through elastic deformation and interface debonding, slowing down crack propagation and reducing the suddenness of failure. The post-peak energy dissipation rate reaches a relatively high level of approximately 1.4–3.0 kW/m^3^ across all particle sizes and contents, about 3–6 times higher than the pre-peak range of 0.3–0.6 kW/m^3^. This demonstrates that cracks propagate rapidly and energy release surges in the post-peak stage, where failure is dominated by energy release associated with crack coalescence.

At 40-mesh, the post-peak energy dissipation rate is relatively high, indicating distinct brittle failure of the R-CTB. Under this condition, the incorporation of rubber particles significantly reduces the energy dissipation rate, demonstrating that rubber addition modifies the failure mode from brittle to ductile. Furthermore, the post-peak energy dissipation rate decreases with reducing particle size, suggesting that fine rubber particles also promote a ductile failure response.

It should be emphasized that the variation in energy dissipation behavior is also closely related to the reduction in cement content. Lower cement dosage leads to decreased elastic energy storage capacity, while rubber inclusion promotes energy dissipation through deformation compatibility. Therefore, the energy evolution reflects a competition between matrix weakening (due to cement reduction) and ductility enhancement (due to rubber addition).

Relevant literatures generally hold that rubber incorporation can improve the energy absorption characteristics of cement-based specimens, but this improvement is mainly attributed to the strength reduction caused by rubber rather than the inherent enhancement of the total energy absorption capacity of specimens [6,50,51,52,53]. In this study, rubber is incorporated by replacing cement; the increase of rubber content is accompanied by the decrease of cement dosage, which further leads to the deterioration of the energy absorption capacity of backfill [54,55,56]. However, the increase of rubber content can transform the backfill from brittle failure to ductile failure, which is consistent with the conclusions of the above literatures.

### 3.4. Analysis of SEM Results

**Figure 19:**
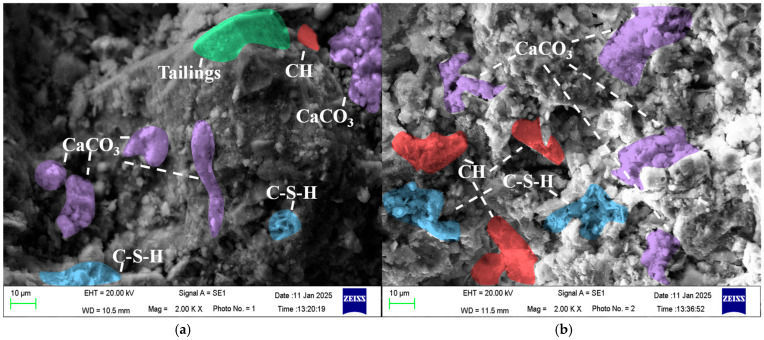

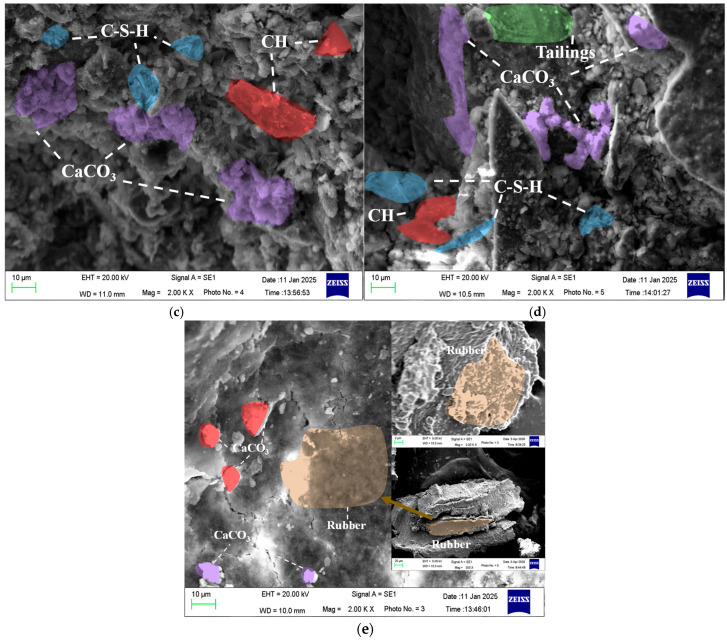
Micromorphology of fracture surfaces of R-CTB: (**a**) 20-mesh + 6%; (**b**) 40-mesh + 2%; (**c**) 60-mesh + 2%; (**d**) 80-mesh + 6%; (**e**) test group.

**Figure 20:**
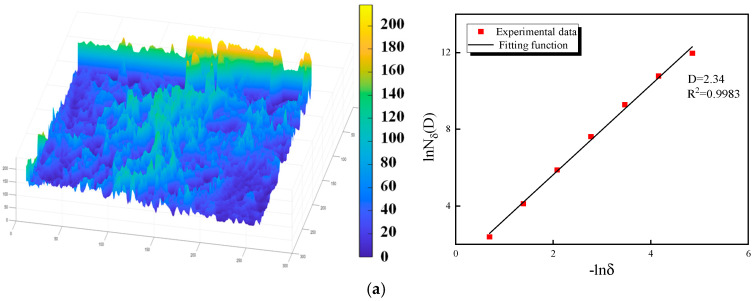

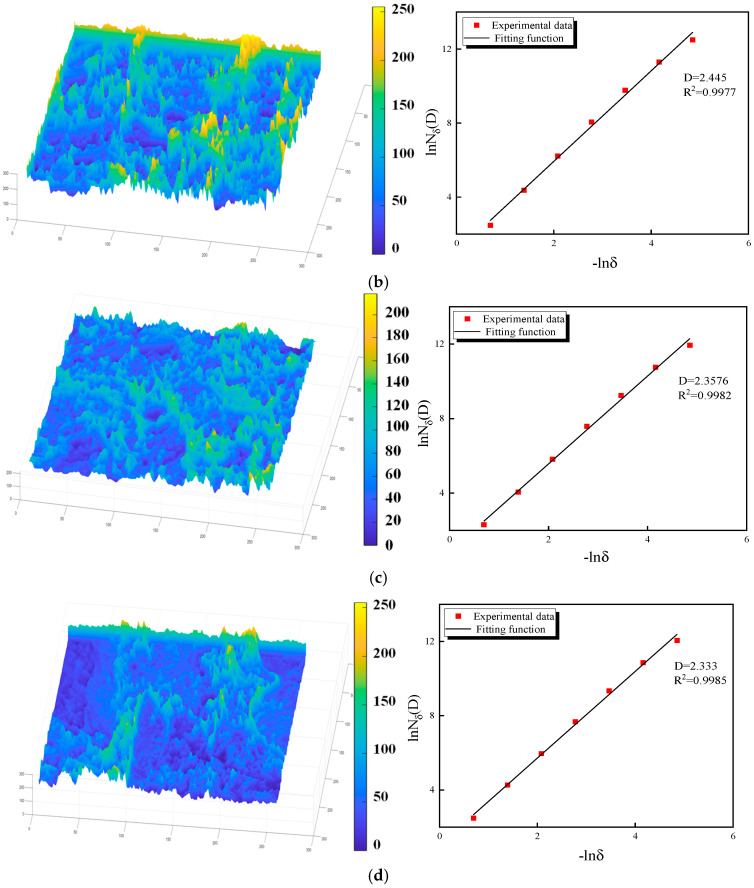
Surface roughness and fractal dimension of fracture surface micromorphology of R-CTB: (**a**) 20-mesh + 6%; (**b**) 40-mesh + 2%; (**c**) 60-mesh + 2%; (**d**) 80-mesh + 6%.

Scanning electron microscopy (SEM) is a widely used technique for characterizing microstructures and hydration products. Figure 19 presents SEM micrographs of the fractured micro-surfaces of representative specimens cured for 28 days, and Figure 20 shows the surface roughness images of the corresponding fracture surfaces. Figure 19a–d’s four groups correspond to 20-mesh + 6% content, 40-mesh + 2% content, 60-mesh + 2% content, and 80-mesh + 6% content, with measured strengths of 2.81 MPa, 4.72 MPa, 3.44 MPa, and 2.7 MPa, respectively. Group e was set as the test group to investigate the reaction state of rubber after the backfill was cured.

Fractal dimension can be adopted to quantitatively describe the geometric complexity of patterns. The three-dimensional box-counting method is a fractal analysis approach for quantifying the complexity and self-similarity of irregular 3D structures, and is suitable for analyzing the microstructural failure morphology of backfill [57]. In this study, the 3D box-counting method was used to quantitatively characterize the complexity of the SEM results.

In the 3D box-counting method, 3D cubes (boxes) of side length δ are used to cover the target 3D structure, and the number of boxes N(δ) containing structural information is counted. According to fractal theory, the side length δ of the box and the corresponding number of covering boxes N(δ) satisfy a power-law relationship, from which the fractal dimension D of the 3D structure can be calculated. The calculation formula is given as follows:

(7) $$D=-\underset{\delta \to 0}{\lim}\frac{\ln N_{\delta }(D)}{\ln\delta }$$

where D is the fractal dimension; δ is the measurement scale; N(δ) is the minimum number of boxes required to cover the target structure.

For the SEM images of R-CTB fracture surfaces analyzed in this study, MATLAB 2025 software was adopted for image preprocessing and fractal dimension calculation. First, the original SEM images were imported into the program, the two-dimensional plane of the image was constructed as the XOY coordinate plane, and the gray values of each pixel in the SEM images were assigned to the *Z*-axis to form a three-dimensional gray-scale surface corresponding to the actual fracture surface topography. On this basis, the 3D box-counting analysis was performed on the gray-scale surface, and the logarithmic fitting of δ and N(δ) was carried out to obtain the 3D box fractal dimension values of the SEM images, which were used to characterize the complexity of the R-CTB fracture surface microstructure.

It can be seen from Figure 19 that the fracture surface of R-CTB with 20-mesh + 6% content exhibits interfacial debonding and pore structures, with uncompleted hydration reaction of raw materials and smooth local areas. The fracture surface of R-CTB with 40-mesh + 2% content is more uniform with fine-scale undulations, and a large amount of hydration products are generated. R-CTB with 60-mesh + 2% content also produces abundant hydration products with a small number of micropores on the fracture surface. R-CTB with 80-mesh + 6% content shows interconnection of multiple microcracks accompanied by slight fine particle spalling, and the fracture surface presents brittle fracture characteristics. As shown in the main figure (2.00 K× magnification) and the magnified views, the cement matrix is dominated by typical hydration products: the layered, flaky structure is calcium hydroxide, and the granular aggregates are calcium carbonate, both of which are the core products of cement hydration. In contrast, the rubber particles exhibit a distinct, smooth, and continuous organic morphology, with clear and sharp interfaces separating them from the surrounding cement hydration products. The interface between rubber and the matrix is a weak physical bonding zone, which is the main reason for the stress concentration and crack initiation in R-CTB, and also confirms the inert nature of rubber in the hydration system.

Figure 20 shows the fractal dimensions of the four types of R-CTB, which are 2.34, 2.445, 2.3576 and 2.333 for the samples with 20-mesh + 6% content, 40-mesh + 2% content, 60-mesh + 2% content and 80-mesh + 6% content, respectively. On the whole, the fractal dimensions of the fracture surfaces of all specimens range from 2.33 to 2.45, indicating that the fracture morphology of R-CTB is between ideal planar fracture and highly complex spatial failure with distinct fractal characteristics. The fracture surface of the 40-mesh + 2% sample has the most complex geometric morphology, the most tortuous crack propagation path, and the highest crack propagation length and fracture energy consumption. This result confirms from the fractal perspective that rubber particles with moderate particle size and low content can effectively exert the functions of crack resistance and energy dissipation. In contrast, when the particle size is too large or too fine and the content is relatively high, the fractal dimension decreases, and the failure mode tends to rapid unstable failure.

## 4. Conclusions

Based on uniaxial compressive strength, elastic modulus, peak strain, comparison between early and long-term curing performance, energy dissipation mechanism, DIC full-field strain analysis and SEM microstructure characterization, combined with the 0% rubber control group for quantitative comparison, this paper systematically investigates the mechanical and failure characteristics of cemented tailings backfill (R-CTB) with various rubber particle sizes and contents, and reveals the influence of rubber particles on the macro-mesomechanical behavior of the backfill. The main conclusions are as follows:

(1) Compared with the control group, all R-CTB groups show lower strength and elastic modulus, and higher ductility. The strength and elastic modulus of R-CTB generally decrease with increasing rubber content, and first increase then decrease with changing rubber particle size. This behavior is not solely attributed to rubber incorporation, but to the combined effect of rubber addition and cement reduction. Since rubber was introduced by partially replacing cement, the decrease in cement dosage reduced hydration product formation (C–S–H) weakened the load-bearing skeleton.
(2) Peak strain analysis shows that the addition of rubber particles and the reduction of particle size can alter the failure mode of R-CTB toward ductile failure; under loading, rubber undergoes elastic deformation and interfacial sliding, which delays crack initiation and propagation. The backfill with 40-mesh rubber particles at low content achieves favorable strength and elastic modulus. By contrast, high rubber content or excessively fine particle size impairs the integrity of the cemented structure, leading to a pronounced degradation in mechanical properties.
(3) The evolution trends of mechanical properties under early (7 d) and long-term (28 d) curing are generally consistent. Prolonging the curing age significantly improves the overall strength of the backfill, yet cannot fully compensate for the inherent strength deterioration caused by high rubber content.
(4) The strength gap between R-CTB and the control group can be narrowed by extending curing time, but cannot be eliminated. Energy evolution analysis indicates that with increasing rubber content and decreasing particle size, the elastic energy storage capacity of specimens declines, the proportion of dissipated energy rises, and post-peak energy release becomes gentler, gradually transforming the backfill failure from brittle to ductile. The reduction in cement content lowers the elastic energy storage limit due to decreased strength. Simultaneously, rubber particles enhance internal friction and interface debonding, which promote energy dissipation. Therefore, the energy conversion process reflects a balance between structural weakening (cement reduction) and ductility enhancement (rubber elasticity).
(5) DIC results show that 40-mesh rubber particles yield the most uniform strain distribution, delayed cracking, and stable stress transfer. Excessively fine particles cause local strain concentration, rapid crack propagation, and premature failure by weakening matrix-rubber bonding. Rubber segregation also induces non-uniform strain, reducing deformation compatibility.
(6) SEM and fractal dimension analysis confirm that rubber particles are chemically inert and do not participate in cement hydration. At low rubber content, the matrix is compact with high fractal dimension; excessively fine particles or high content increase pores, reduce structural integrity and fractal dimension, and cause unstable failure.

However, the coupled effects of particle size and content on R-CTB behavior remain unclear, limiting the accurate interpretation of failure mechanisms. Future work will focus on long-term durability, dynamic mechanical properties, and rubber interface modification to further optimize backfill performance.

## Figures and Tables

**Table 1:** Main chemical compositions of tailings.

Component	SiO_2_	Al_2_O_3_	K_2_O	CaO	Fe_2_O_3_	Na_2_O	MgO	SO_3_
**Content/%**	62.27	17.61	6.59	5.18	3.83	2.01	1.79	0.42

**Table 2:** Main chemical compositions of cement.

Component	CaO	SiO_2_	Al_2_O_3_	Fe_2_O_3_	SO_3_	MgO	K_2_O	TiO_2_
**Content/%**	55.53	23.52	10.26	3.93	2.70	1.50	1.07	0.50

**Table 3:** Mix proportion design of experimental materials.

Curing Age	Tailings (%)	Cement (%)	Rubber Particles (%)	Rubber Particle Size (Mesh)
7 d/28 d	80	18	2	20
				40
				60
				80
		16	4	20
				40
				60
				80
		14	6	20
				40
				60
				80
		12	8	20
				40
				60
				80

**Table 4:** Energy of R-CTB at each loading point under different rubber contents and particle sizes.

Rubber Particle Size (Mesh)	Rubber Particle Content	Energy at Each Loading Point (KJ/m^3^)														
		A			B			C			D			E		
		*U_T_*	*U_E_*	*U_D_*	*U_T_*	*U_E_*	*U_D_*	*U_T_*	*U_E_*	*U_D_*	*U_T_*	*U_E_*	*U_D_*	*U_T_*	*U_E_*	*U_D_*
0	0	7.19	0.79	6.4	15.94	8.54	7.39	30.12	21.39	8.73	36.79	23.43	13.35	74.58	10.4	64.14
20	2%	5.73	0.92	4.8	14.72	6.45	8.26	27.28	16.36	10.92	29.75	16.85	12.99	89.4	6.09	83.31
	4%	6.09	1.01	5.08	12.83	6.75	6.07	28.35	17.04	11.31	30.2	17.26	13.05	80.02	6.16	73.86
	6%	6.59	1.03	5.56	11.51	4.88	6.63	21.61	12.37	9.23	24.48	12.74	11.74	74.12	4.60	69.51
	8%	5.97	1.11	4.86	11.78	5.82	5.97	23.2	14.8	8.4	28.44	15.31	13.24	75.72	5.44	70.27
40	2%	7.54	0.65	6.9	13.53	6.31	7.22	26.54	17.9	8.64	35.99	20.14	15.84	119.4	7.24	111.8
	4%	7.45	0.83	6.62	12.83	5.51	7.32	23.9	14.05	9.85	26.33	14.57	11.75	82.09	5.24	76.84
	6%	6.35	0.88	5.47	13.24	5.62	7.62	22.37	14.9	7.46	26.35	16.49	9.86	105.5	7.22	98.35
	8%	6.13	0.76	5.36	11.67	5.63	6.03	24.66	14.82	9.83	29.28	15.52	13.76	99.19	5.58	93.60
60	2%	5.88	0.9	4.97	12.32	6.16	6.16	25.93	16.4	9.52	29.92	17.2	12.72	80.17	6.16	74.0
	4%	5.66	0.87	4.79	12.54	6.63	5.91	28.18	18.81	9.36	32.0	19.74	12.26	101.5	7.13	94.36
	6%	7.88	1.80	6.07	18.23	10.4	7.81	37.14	26.62	10.52	46.34	28.74	17.59	102.5	10.3	92.2
	8%	6.76	1.6	5.16	12.9	7.05	5.84	25.76	16.77	8.99	31.61	18.23	13.38	76.84	6.56	70.28
80	2%	7.23	1.6	5.63	18.11	10.9	7.12	49.68	28.6	21.08	56.55	29.29	27.26	128.2	10.4	117.6
	4%	5.93	1.11	4.81	10.78	4.64	6.13	18.51	10.75	7.76	22.55	11.47	11.07	45.72	4.08	41.63
	6%	6.28	1.39	4.88	12.4	6.16	6.24	25.27	15.1	10.16	29.58	15.92	13.66	69.76	5.73	64.03
	8%	5.77	1.5	4.26	13.46	7.73	5.72	25.73	16.07	9.66	31.09	16.83	14.25	67.9	6.02	61.88

## Data Availability

The raw data supporting the conclusions of this article will be made available by the authors on request.
